# Optimization of Reinforcement Schemes for Stabilizing the Working Floor in Coal Mines Based on an Assessment of Its Deformation State

**DOI:** 10.3390/ma18133094

**Published:** 2025-06-30

**Authors:** Denis Akhmatnurov, Nail Zamaliyev, Ravil Mussin, Vladimir Demin, Nikita Ganyukov, Krzysztof Zagórski, Krzysztof Skrzypkowski, Waldemar Korzeniowski, Jerzy Stasica

**Affiliations:** 1 Department of Information and Computing Systems, Abylkas Saginov Karaganda Technical University, 56 Nursultan Nazarbayev Avenue, Office 406, Karaganda 100027, Kazakhstan; d.akhmatnurov@gmail.com (D.A.); r.a.mussin@mail.ru (R.M.); vladfdemin@mail.ru (V.D.); nikitagd99@mail.ru (N.G.); 2 Faculty of Mechanical Engineering and Robotics, AGH University of Krakow, Mickiewicza 30 Av., 30-059 Kraków, Poland; 3 Faculty of Civil Engineering and Resource Management, AGH University of Krakow, 30-059 Kraków, Poland; skrzypko@agh.edu.pl (K.S.); walkor@agh.edu.pl (W.K.); stasica@agh.edu.pl (J.S.)

**Keywords:** coal-bearing rock mass, stress–strain state, mine workings, anchor support, floor heaving, geomechanical modeling, underground stability, rock stratification, near-contour deformations, reinforcement technology

## Abstract

In the Karaganda coal basin, deteriorating geomechanical conditions have been observed, including seam disturbances, diminished strength of argillite–aleurolite strata, water ingress, and pronounced floor heave, all of which markedly increase the labor intensity of maintaining developmental headings. The maintenance and operation of these entries for a reference coal yield of 1000 t necessitate 72–75 man-shifts, of which 90–95% are expended on mitigating ground pressure effects and restoring support integrity. Conventional heave control measures—such as relief drifts, slotting, drainage, secondary blasting, and the application of concrete or rock–bolt systems—deliver either transient efficacy or incur prohibitive labor and material expenditures while lacking unified methodologies for predictive forecasting and support parameter design. This study therefore advocates for an integrated framework that synergizes geomechanical characterization, deformation prognosis, and the tailored selection of reinforcement schemes (incorporating both sidewall and floor-anchoring systems with directed preloading), calibrated to seam depth, geometry, and lithological properties. Employing deformation state assessments to optimize reinforcement layouts for floor stabilization in coal mine workings is projected to curtail repair volumes by 30–40% whilst significantly enhancing operational safety, efficiency, and the punctuality of face preparation.

## 1. Introduction

In the coal mines of the Karaganda basin, ensuring the stability of development (main) workings has become increasingly critical year by year due to the progressive deterioration of geo-engineering conditions—seam disturbances, reduced rock mass integrity, water ingress, and the like. Across the basin, the average labor intensity required to maintain workings for every 1000 t of coal extracted reaches 72–75 man-shifts, yet only 15–17% of the total tunnel length preserves acceptable cross-sectional dimensions [1].

Extensive in situ observations within mine workings have demonstrated [2] that both the intensity and absolute magnitude of heave movements depend on a multitude of geo-engineering, technological, and operational factors. To date, the fundamental mechanisms driving floor heave remain incompletely understood; neither the principal governing laws have been fully elucidated, nor have reliable calculation and forecasting methodologies been developed, and no substantiated, practicable countermeasures have been defined for varying mining depths, roadway types, or the physical–mechanical and strength properties of host strata [3,4].

Accordingly, formulating and developing a predictive and evaluative method for soil–rock heave, together with effective mitigation measures—tailored to the specific geo-engineering and mining–technical conditions of the Karaganda coal basin—is a vital scientific and technical challenge for preserving panel headings at various depths during intensive extraction of gently inclined seams [5,6].

During coal deposit development, host rock deformations frequently manifest as inward extrusions—heaves—most pronounced on the roof and floor of development workings. Owing to the seams’ genesis, immediate roof and floor strata are typically weaker than overlying and underlying units and often contain shear zones produced by differential compaction and subsequent tectonic reworking [7,8]. Heave of floor strata severely complicates mining operations: empirical evidence indicates that when displacements exceed 300–350 mm, specialized countermeasures become indispensable [9,10].

Field studies further confirm that heave displacement magnitudes are governed by a complex interplay of geological and operational parameters. Effective mitigation is achieved by unloading or stabilizing the host rock mass surrounding the excavation from prevailing stress fields. Proposed unloading techniques include driving relief drifts, creating slot cuts or boreholes, and pre-blasting to generate fragmentation zones—measures that, however, entail intricate procedures, high labor demands, and significant costs.

Consequently, the predominant practice in operating coal mines remains reactive: blasting of heaved strata is undertaken only to restore clearance, addressing symptoms rather than root causes. This yields only transient, marginal benefits, while long-term expenditure on materials and labor for heave control remains substantial.

Recently, the technical literature has reported successful deployment of high-capacity steel–polymer rock–bolt support systems to counteract floor heave [11,12]. Nevertheless, these accounts lack a mechanistic rationale for the observed stabilization effect, do not furnish calculation methodologies for design of anchor parameters and layouts, nor delineate the applicable scope of this approach.

This study’s research objectives are as follows: to investigate the strength characteristics and structural features of the rock mass; to develop innovative, combined support technologies employing contour anchorage systems; and thereby to ensure operational safety, enhance economic efficiency, and improve the reliability of underground workings under complex geo-engineering conditions. Through an integrated influence on the rock mass’s structure, strength, and physical–mechanical properties, this research aims to achieve a consolidation effect that stabilizes the support conditions of the host strata.

## 2. Materials and Methods

In the contemporary literature, the critical importance of incorporating geological and geomechanical parameters into the analysis of floor heave in mine workings—particularly with increasing mining depth—is emphatically underscored. For example, Gong et al. demonstrated that, at depths exceeding 1000 m in weakly cemented argillite–aleurolite sequences, post-extraction stress redistribution induces pronounced heave immediately ahead of the face [13]. Subsequent experiments by Sun et al. revealed that moisture uptake in clay horizons can halve their strength characteristics, thereby markedly accelerating deformation processes in the floor [14]. Similarly, Li et al. and Xie et al. have shown that soft, friable strata and poorly sealed coal seams are especially susceptible to extreme heave owing to the absence of dependable floor support [15,16,17]. Moreover, Xu et al. established a clear dependency of heave magnitude on roadway geometry and the distance from the advancing face, confirming the necessity of integrating spatial variables into predictive models [18].

To minimize and control floor heave, several technological strategies—validated both in the field and by numerical simulation—have been proposed by Chen et al. and Zhao et al. [19,20]. Of particular interest is the integration of anti-shear piles, which stabilize the flank of the rock mass, with a combined system of lateral rock–bolt reinforcement and cement grouting, as described by Sakhno et al. and Shang et al. [14,21]. The hybrid application of concrete blocks and steel piles, in conjunction with fully mechanized backfilling, has proven highly effective by redistributing stresses and reducing mass porosity [21,22,23,24]. Under conditions of competent roof strata, the adoption of roof-cutting techniques has successfully attenuated vertical loading on the floor by segmenting the overlying rock block [25,26].

Additional design tools include floor classification schemes and high-density drainage drilling, as proposed by Li et al. and Xu et al., which enable pre-development stress relaxation and quantitative assessment of heave potential [27,28]. In the present study, our methodology for controlling floor heave is founded upon the integration of these technologies within a comprehensive finite-element modeling framework.

**Figure 1:**
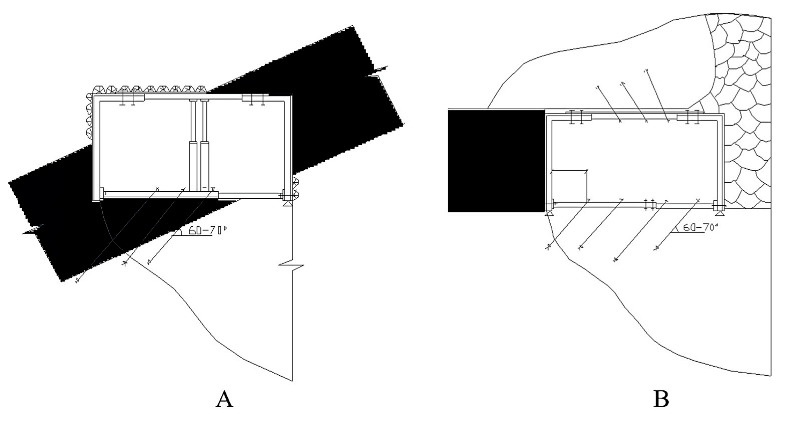
Reinforcement layout for the entry both ahead of and behind the longwall face. (**A**) Optimized scheme for installing anchors in both the roof and floor of the entry, located immediately ahead of the longwall face and in relation to the mined-out void. (**B**) Schematic comparison of preparatory headings in weak floor conditions using an enhanced lateral anchorage system within the surrounding rock mass.

Figure 1 presents the reinforcement specification for a roadway immediately behind the coal face, illustrating an optimized anchor installation scheme in both roof and floor relative to the excavated volume. It also offers a schematic comparison of development headings in weak floor conditions: (a) employing conventional vertical rock bolts inclined at 60–70° to the seam vs. (b) an enhanced lateral anchorage system within the surrounding rock mass.

**Figure 2:**
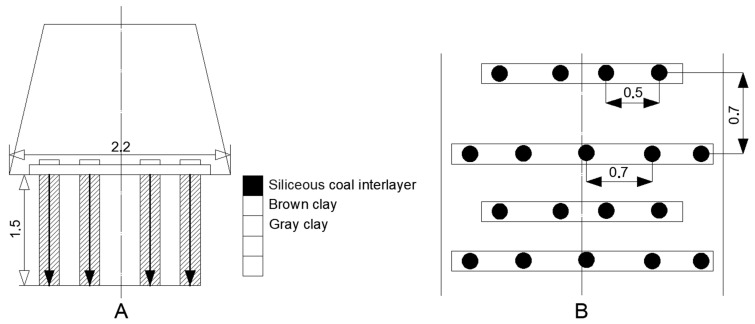
Technological schemes of anchor soil reinforcement in preparatory workings (practices in Ukraine, the United Kingdom, and Germany). (**A**,**B**) Cross-section and plan view, respectively, of the floor reinforcement system; anchor installation scheme in the mine floor (haulage drift) of the “Butovskaya-Donetskaya” mine.

The figure below depicts the technological schemes of soil reinforcement applied in mining practice in Ukraine, the United Kingdom, and Germany (Figure 2), alongside a geotechnical model of the reinforcement zone of the excavated floor.

(A) Cross-section illustrating the stratification of the soil, comprising a sequence of gray clay, brown clay, and siliceous coal seams, with anchor elements installed at 0.3 m intervals.
(B) Reinforcement plan showing the spatial layout of anchor boreholes with longitudinal and transverse spacings of 0.7 m and 0.5 m, respectively.

This scheme elucidates the interaction between the inherent geological heterogeneity and the designed anchoring grid.

**Figure 3:**
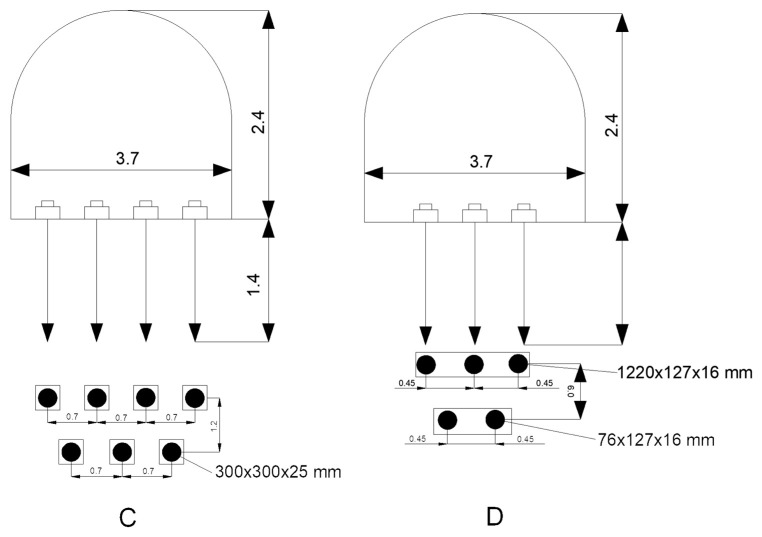
Comparison of soil reinforcement schemes in mine workings: distributed (**C**) and concentrated (**D**) anchor systems.

Figure 3 illustrates a comparison of soil reinforcement schemes in mine workings:

(C) The layout employing concrete slabs (300 × 300 × 25 mm), arranged in a grid with 0.7 m spacing in both directions, exemplifies the standard ground support in weak rock conditions.
(D) Soil reinforcement using steel baseplates measuring 1220 × 127 × 16 mm and 76 × 127 × 16 mm, installed at 0.45 m intervals, reflects the geometric configuration and relative effectiveness of the different reinforcement systems in controlling ground deformations under concentrated loads.

Figure 3C,D denote, respectively, the cross-section and plan of a soil reinforcement system employing distributed and concentrated anchor layouts; this illustrates the anchor reinforcement technology applied at the Cordovan Mine (Scotland).

In mine workings unaffected by longwall extraction (first deformation type), deformation of the soil layers is limited to a minor bulging (see Figure 2), the magnitude of which remains below the rock’s relative longitudinal strain (15 × 10^−3^m). Complete attenuation of these deformations is typically observed over a period of 150–300 days. A comparison of lateral displacements across the width of the heading indicates that the intensity of soil deformation is somewhat greater at the center of the drift than at its flanks. Figure 3 presents the relative deformations of the soil layers over time, measured from the excavation boundary into the rock mass.

**Figure 4:**
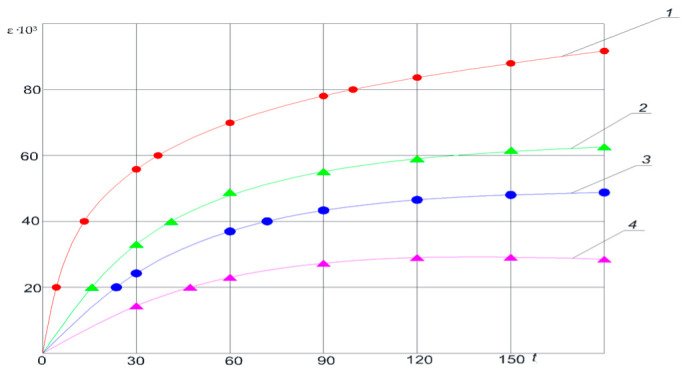
Relative deformations of the soil layers over time, measured from the excavation boundary into the rock mass. 1—up to 1 m; 2—1–2 m; 3—2–3 m; 4—3–4.5 m.

The accumulation of soil deformations over time under different reinforcement schemes shows that, in unreinforced soil (curve 1), deformations grow most rapidly; the use of conventional concrete elements (curve 2) yields a noticeable but relatively moderate reduction in deformation; the steel plate system (curve 3) more effectively restrains ground movements; and the greatest suppression of deformations over the 150-day observation period was achieved by the force-expanding modules (FEM, curve 4). Figure 4 presents the schematic layout of deformation zones in a drift composed of weak rock strata.

**Figure 5:**
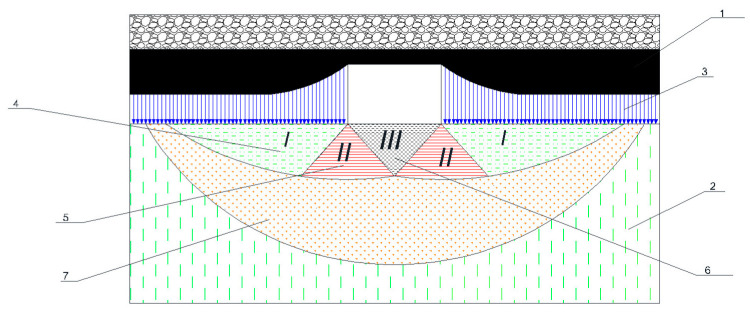
Distribution of deformation zones in the soil of the drift composed of weak rock strata: 1—overburden rocks; 2—intact host rock; 3—excavation contour; 4—Zone I: elastic deformation; 5—Zone II: plastic failure zone; 6—Zone III: extrusion zone; 7—reinforcement zone (steel–polymer anchors).

In Figure 5, the numbered zones are as follows: 1 denotes the coal seam; 2 represents the weak rock strata; 3 indicates the soil deformation zone; 4 marks the active rock-pressure zone; 5 corresponds to a transitional zone with slip lines; 6 shows the passive pressure zone; and 7 identifies the elastic deformation zone.

### 2.1. Theoretical Aspects of Soil Heave in Coal Mine Workings

Water saturation of mine workings has a profound effect on the intensity of soil heave. Dense clays and argillaceous shales, when fully saturated, lose between two and ten times their original strength. As the depth of mining increases, the intensity of heave likewise rises, and this trend becomes particularly pronounced beyond certain depths. Up to approximately 1000 m below surface, the relationship between depth and heave magnitude is almost linear. For each coal seam and its surrounding strata there exists a critical depth beyond which heaving processes become self-evident.

In preparatory headings within the influence zone of longwall extraction, the intensity of soil heave increases as the face advances toward the observation point, reaching a peak shortly before the face passes. Once the face has passed and retreats, heave intensity gradually diminishes and asymptotically approaches a stable residual value. In isolated workings, heave generally progresses monotonically over time and attenuates gradually as the ground support system stabilizes.

In gate roads adjacent to the coal seam—where the roof and floor are composed of laminated argillites and siltstones—the heave and degradation process unfolds in several stages. Initially, delamination occurs along bedding planes without rupturing individual layers (Figure 5. Next, those layers disintegrate into blocky fragments, giving rise to multi-hinged arching beneath the roof (Figure 5). Subsequently, the degraded material is expelled laterally, pressing into the working void (Figure 5). Finally, as illustrated in Figure 5, the excavation contour is stabilized using steel–polymer anchor bolts.

**Figure 6:**
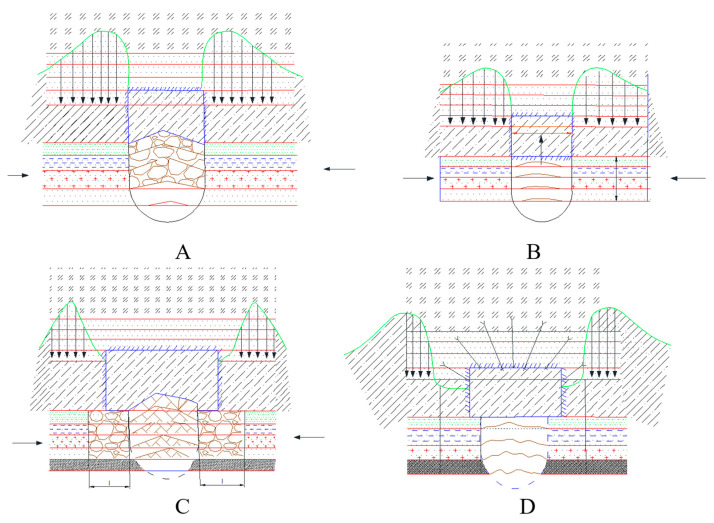
Stages of soil heave in mine workings: (**A**) Initial stress concentration and formation of unloading zones near the excavation boundaries; (**B**) Development of a subsidence trough due to progressive collapse of the roof strata; (**C**) Expansion of the plastic deformation zone into the weak floor rocks under the influence of horizontal stress; (**D**) Stabilization of the excavation contour through backfilling and application of artificial reinforcement systems.

Figure 6 depicts the zoning of heave deformation around the excavation void, showing the depth of delamination under the combined action of horizontal and vertical rock-pressure components within the inelastic deformation, failure, and extrusion zones. The figure also indicates the direction and magnitude of the migration of the maximal deformation zone into the rock mass through the successive stages of the deformation process when the contour is reinforced with steel–polymer anchoring.

An analysis of the stress distribution in Figure 6 shows that, for an unsupported base (curve 5), the highest stress concentrations are located directly at the interface between the excavation and the rock mass, leading to the rapid onset of plastic deformations within the weak argillite horizons.

The application of conventional concrete (or metallic) elements (curve 7) transfers part of the load to the upper support contour, thereby slightly unloading the soil; however, zones of high stress shift only marginally. In contrast, the use of a prestressed steel plate (curve 6) achieves a more uniform redistribution of loads, reducing peak stresses and spreading them over a larger cross-sectional area. The most effective reinforcement proved to be the force-expanding modules (curve 4): their outward thrust creates a supporting “shell” contour that extends deeply into the rock mass, substantially lowering the stress concentration factor in the near-surface zone and decelerating the development of heave.

**Figure 7:**
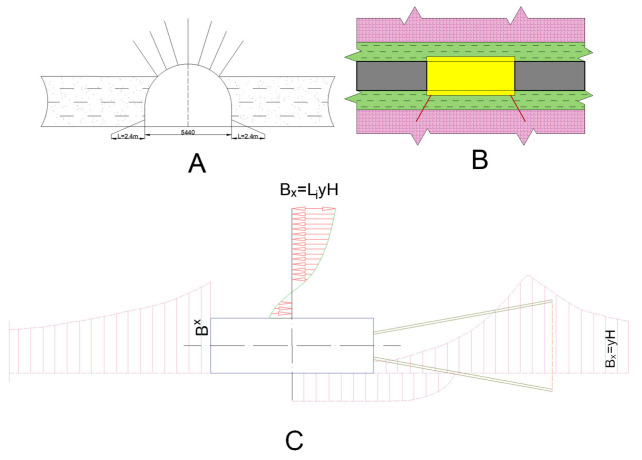
Effective soil reinforcement scheme from the excavation contour to mitigate heave. (**A**,**B**) correspond to the arch-shaped and rectangular borehole anchoring schemes for soil reinforcement in mine workings; (**C**) is the deformation profile for scheme (**B**).

Figure 7 depicts an optimized soil reinforcement scheme relative to the excavation boundary, designed to permit drilling with standard rotary rigs, facilitate the removal of drilling cuttings, and locate anchors within the pressure-bearing zone around the excavation. This configuration involves enlarging the borehole mouth—leaving it intentionally void of grout—to relieve lateral rock strata in this region, while the remainder of the hole (standard 28 mm diameter) is fully grouted to secure the anchor.

**Figure 8:**
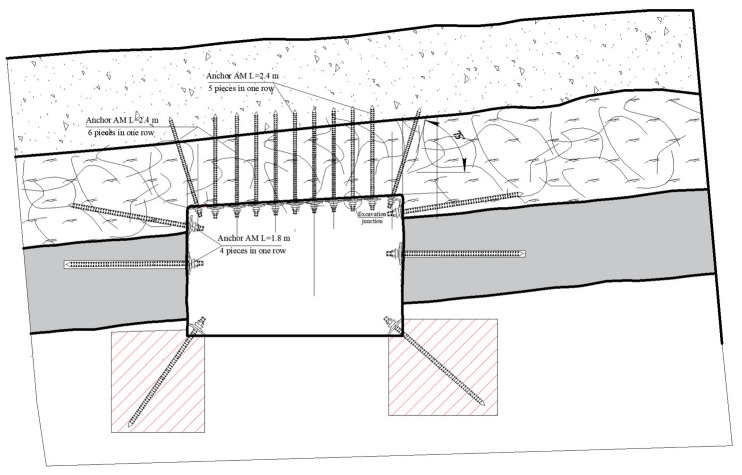
Installation method for near-floor anchors along the sidewalls of the excavation.

To quantify stress concentration factors more precisely and validate the analytical findings, numerical simulations were conducted using the finite difference method in FLAC2D (Figure 8). The modeling results confirmed the efficacy of the proposed schemes: the peak stress state coefficient adjacent to the excavation contour was reduced by 35% compared to the baseline case and was virtually absent in the upper portion of the argillite horizon.

### 2.2. Technological Solutions for Soil Reinforcement and Their Investigation

With an increase in the bulk density of the roof strata, the depth of the soil heave zone in mine workings generally diminishes. Conversely, the greater the bulk density of the sidewall strata and the height of the working walls, the deeper the heave zone propagates into the soil.

A specialized anchoring technology has been developed to mitigate soil heave under diverse mining–geological conditions, employing various support types and accounting for all complicating factors associated with excavation and the prevailing geomechanical environment during driving and operation of the workings.

This technology envisages the installation of near-floor anchors along the sidewalls of the excavation. Inclined boreholes for these anchors are drilled at angles of 20–40° to the vertical. Borehole length is determined by the technical capabilities of the drilling rig and is typically 1.6, 2.4, or 2.9 m. Anchors are arranged in a staggered pattern, perpendicular to the bedding planes of the soil strata.

**Figure 9:**
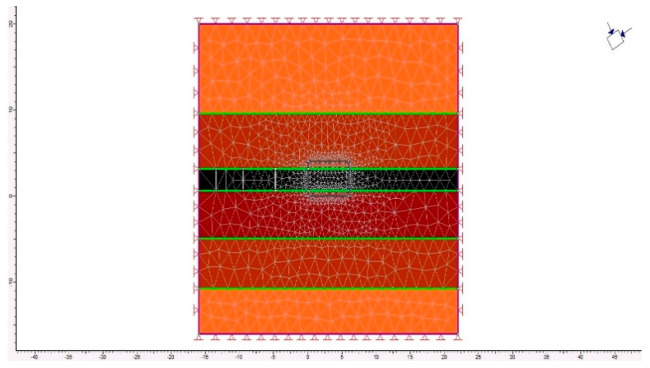
Geomechanical model of the near-contour rock mass surrounding the excavation, illustrating the geometrical and material parameters of the coal seam and enclosing strata.

After importing the geomechanical model into the software environment, it was discretized into finite elements. Across the entire domain, the maximum element size was set to 8 m, while in zones expected to experience peak loads, the mesh was refined to a 2 m grid spacing. This configuration ensured a uniform representation of each lithological layer in accordance with its physical properties. At the microscale, the mesh was generated using the program’s built-in meshing tools (Figure 9).

The investigation of the stress–strain state of the rock mass was carried out for the seams of the Karaganda coal basin. Figure 9 presents the predicted soil heave value (P_f_) in the excavation as a function of the ratio of anchor length (Lₐ) to excavation width (Bᵦ).

A field production trial of near-floor, off-contour anchors was conducted at the “Kazakhstanskaya” mine in the Karaganda coal basin. Anchors were installed in the conveyor drift 334D6-1-V (Kazakhstanskaya mine), in the upper seam layer of the thick D6 bed, at anticlinal and synclinal stations PK 104–103. The conveyor drift, supported by a mixed system of steel arches and seven resin-grouted bolts at 0.75 m spacing, remained relatively stable ahead of the longwall face, which had advanced 300 m from the gate. The main roof collapsed in 90 m intervals.

In contrast, the ventilation drift 334D6-1-V—excavated adjacent to the overlying D7 seam’s gas drainage gallery—experienced significant deformation of both floor and roof where steel arches were spaced at 0.75 m; tighter arch spacing (0.5 m) produced a more stable opening. An isolating coal pillar 2.5 m wide was left between the conveyor and ventilation drifts.

Anchors were installed on both the ribs and off-rib sidewalls, 0.5 m from the arch frames, using a “SuperTurbo” drill capable of penetrating 1.0–1.6 m per hole. Drilling utilized 1.2 m two-bladed and 1.8 m/2.2 m three-bladed rods; operations later standardized on the longer rods to accelerate progress. Holes were drilled at 45° to both the contour and the drift axis; spoil was cleared from each 0.2–0.4 m pilot hole to prevent resin slug contamination. Drilling and anchoring required eight minutes per bolt, with 0.1–0.2 m spacing between holes and up to 0.5 m resin overlap in the soil.

Each anchor comprised a 2.4 m twisted-steel tendon grouted with three resin capsules—one fast-setting (0.35 m, 15–20 s set time) and two slow-setting (0.6 m each, 2 min set time); later trials used only the slow capsules. Tendons were torqued into the resin-filled holes via an adapter on the drill and locked with a nut once the shear pin fractured. The expanding resin matrix consolidated the surrounding strata, forming a reinforced floor contour and shifting the pressure peak 1.0–2.0 m deeper into the rock mass.

**Figure 10:**
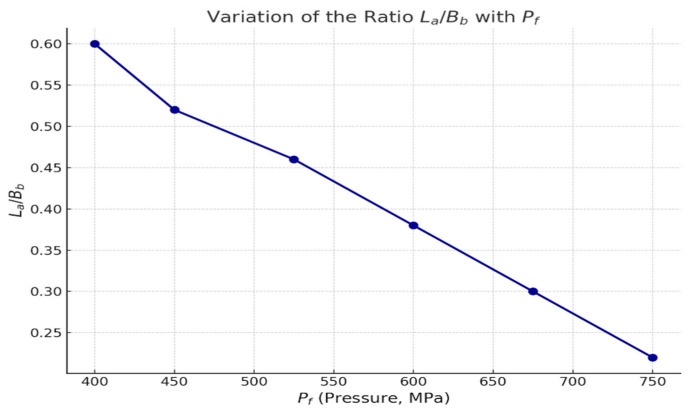
Predicted soil heave (P_f_) as a function of the ratio of anchor length (Lₐ) to excavation width (Bᵦ).

Anchor clusters (pairs of off-contour near-floor bolts) were positioned every 1.5 m along both ribs (Figure 10). Monitoring over three months recorded uneven floor heave—most severe on the right rib—measured at 18 mm in month 1, 11 mm in month 2, and 9 mm in month 3 (Figure 10). The primary driver of heave was the presence of weak argillite layers, 1–16 m thick, immediately beneath the drift. Field data further demonstrated a direct correlation between the degree of roof strata delamination in the longwall pressure zone and the width of the adjacent gate road: greater roadway width produced more extensive delamination. The physical and strength parameters of the coal-bearing rock mass used to develop the subsequent geomechanical model are summarized in Table 1.

**Figure 11:**
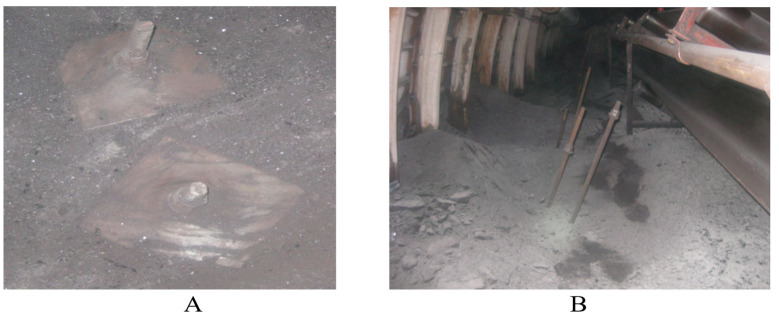
Clusters of near-floor off-contour anchors installed along both sides of the studied mine working. (**A**)—Installation of anchors in the floor on the non-entry side. (**B**)—Installation on the entry side at an angle outside the excavation contour.

In the view shown in Figure 11A, a section of the mine floor is captured immediately after the removal of the wooden anchor formwork: two steel bearing plates (1220 × 127 × 16 mm) are visible, with fine-grained spoil and argillite fragments heaped on and around them. The center of each plate is occupied by the shaft of an anchor bolt, generously coated with epoxy resin, indicating that the anchors were installed and secured in the rock mass very recently.

In the view shown in Figure 11B, the same working is seen from a wider perspective: on the right, the conveyor belt is visible, and behind it, the arched steel support. Immediately adjacent to the conveyor, three inclined anchors are mounted in the floor, their upper ends protruding above the surface and held in the heads of steel couplings. Judging by the appearance of the resin and the fresh chips in the argillite, the drilling and anchor-installation works were completed only shortly before the photograph was taken.

**Figure 12:**
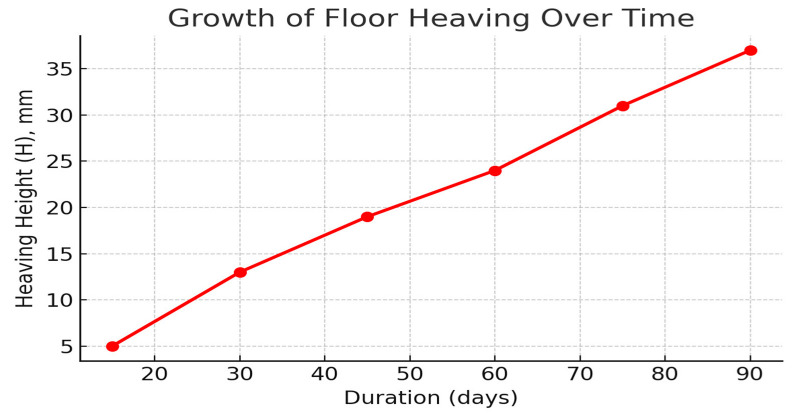
Dynamics of soil heave development in the mine working.

The dynamics of floor heave development during the observation period are illustrated in Figure 12.

Analytical modeling of the placement schemes for near-floor anchors, based on field observations at the mine, was carried out using the FLAC2D software v8.0 (Itasca Consulting Group, Inc., Minneapolis, MN, USA) under various mining–geological conditions of the Karaganda coal seam workings. The results demonstrated that the greatest reinforcement effect is achieved in rectangular section drifts when roof bolt support is combined with a hybrid side-anchoring scheme.

**Figure 13:**
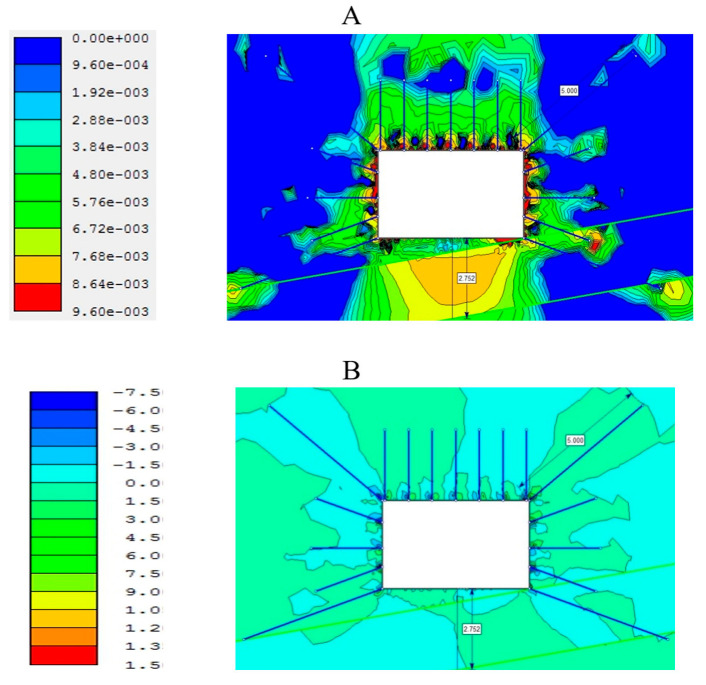
Development of deformations ((**A**): **left**—displacement scale; **right**—deformation pattern) and shear stresses ((**B**): **left**—stress scale; **right**—stress distribution) in the soil with 5.0 m long upper side anchors and 5.0 m long near-floor anchors.

In this configuration, the upper side anchor—typically installed at greater depth—is located in the rock pressure zone, outside the excavation contour, within the enclosing strata. Its purpose is to shift the stress concentration peak deeper into the rock mass, beyond the deformation zone induced by the excavation. The lower, deeper side anchor is positioned so as to inhibit a lateral propagation and extrusion of the sidewall strata into the drift floor (see Figure 13) [23,24,26,29,30].

Thus, it can be concluded that deformation and stress distribution in both the sidewall strata and the drift floor are influenced primarily by the side anchors rather than the near-floor anchors. This scheme also contributes to reduced deformation of the surrounding rock mass and helps to minimize gas emission from the coal seam.

**Figure 14:**
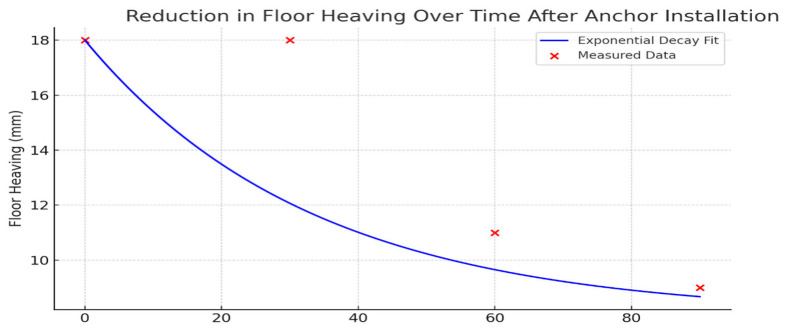
Dynamics of soil heave reduction following the installation of side anchors.

Figure 14 illustrates the reduction in soil heave over time following the installation of side anchors.

## 3. Results

The experimental and numerical work carried out within the framework of this study has revealed a clear interrelationship between the geometry of the soil and side rock support systems and the development of the stress–strain state of the coal-bearing massif. Field measurements showed that the decisive role in limiting soil heave and reducing deformations around the excavation contour is played not by near-floor anchors, but by lateral anchors strategically placed outside zones of stress concentration.

These conclusions were consistently confirmed both by in situ observations and by finite difference modeling in the FLAC2D program v.8.0.

One of the most illustrative results was obtained during a three-month monitoring campaign at the “Kazakhstanskaya” mine: it was found that soil heave decreased from 18 mm in the first month to just 9 mm in the third month after the installation of paired clusters of lateral anchors. The reduction in heave followed an almost exponential decay curve, indicating a stabilizing effect as the support contour shifted deeper into the massif.

Moreover, numerical modeling made it possible to assess the interaction of various support elements with the deformation zones. The combined action of upper and lower lateral anchors forms a stabilizing shell, altering the stress trajectories from the excavation floor and thus preventing both shear failures and the formation of tensile cracks in weak argillites [31,32].

The analysis also showed that increasing the ratio of anchor length to excavation width (Lₐ/Wₑ) leads to a nonlinear reduction in the predicted soil heave, with optimal values achieved at ratios greater than 1.5. This confirms the recommendation to use extended anchors when working in weak rock under high horizontal stresses [20].

The relationship between the predicted heave magnitude (G) of the excavation floor and the ratio of anchor length (L) to excavation width (W) is described by the following equation, with the anchor length to be installed in the excavation floor determined by the empirical formula [33]:

(1) $$\begin{array}{c} L=\frac{K_{i}\cdot B\cdot G}{P},m \end{array}$$

where $K_{i}$—empirical coefficient (for the Karaganda coal basin, it is equal to 6.75);

B—width of the excavation (in the clear), m;

G—magnitude of soil heave, m;

P—compressive strength of the soil rocks, MPa.

The installation spacing of floor anchors is recommended to be twice the number of metal arch supports per linear meter of the excavation.

## 4. Discussion

The development of floor heave in coal mine workings in the Karaganda basin is driven by a combination of geological and technical–technological factors, among which the key ones are the disturbed bedding of argillite–aleurolite layers, their low strength under high water saturation, and the wide profile of preparatory roadways [1,2]. Classical models of anisotropic behavior in fractured rock masses, as described by Wittke [3] and Jaeger et al. [4], allow for the prediction of stress concentration zones. However, practical studies by Sakhno et al. [13] have demonstrated that the use of anti-shear piles in the sidewalls of workings significantly alters the redistribution of shear stresses in the near-surface strata.

The choice of a combined scheme of lateral and floor anchoring is based on experimental data from Gong et al. [18] and Chen et al. [19], which show that only when a rigid “shell” and floor bracing elements are formed simultaneously is it possible to achieve a noticeable reduction in heave by shifting peak loads to a depth inaccessible to the direct extrusion of weak argillite layers. Specifically, field observations recorded a reduction in heave from 18 to 9 mm over three months at an established anchor length to excavation width ratio L/B > 1.5, which corresponds to the recommendations of Li et al. [15] and Xie et al. [16].

Numerical modeling in FLAC2D, carried out using the methodology of Ma and Zoback [5] and the Mohr–Coulomb criterion, confirmed that at lateral anchor installation angles of 20–40° and depths of 1.6–2.9 m, the zone of maximum shear and normal stresses moves away from the excavation contour by 1–2 m, significantly slowing the development of plastic deformations and preventing the formation of slip along bedding planes [23]. Uniform, rigid support achieved with stiff power-expanding modules (SPMs) demonstrated an effectiveness comparable to that of concrete and steel elements with minimal labor costs, consistent with the conclusions of Latypov et al. [6] and Sun et al. [21].

The introduction of geomechanical zoning in the design of preparatory workings, based on the criteria of Zhabko [8] and Kashnikov [7], as well as modern floor stability classification systems proposed by Li [27] and Xu, Q & Zhou [28,34], allows for the justification of anchor length and spacing, as well as the identification of priority areas for reinforcement. An integrated approach combining field monitoring, laboratory testing, and numerical modeling ensures a significant reduction in repair work volumes (by up to 30–40%) and increases the reliability of preparatory workings in the challenging geological and technical conditions of the Karaganda basin.

## 5. Conclusions

The identified patterns of changes in the stress–strain state of the coal-bearing rock mass (displacements, stresses, and failure zones), depending on key mining–geological and mining–technical factors and mining conditions, make it possible to determine the optimal support parameters for operational conditions, thereby enhancing the stability of the floor contours of preparatory mine workings.

The necessity of developing and improving technologies for an effective and safe reinforcement of near-surface rocks during the driving of mine workings in gently dipping and inclined coal seams has been substantiated. It can be concluded that deformations and stresses—both in the side rocks and in the floor of the workings—are most significantly influenced by lateral anchors rather than floor anchors.

The reduction in the cost of mine drivage when using directional support systems (by 15%) (or mass weakening, depending on operating conditions) at the mines of the Karaganda coal basin is achieved due to savings in financial resources (14–17%), reduced labor costs (10–15%), increased drivage rates (20–27%), and safer mining operations.

## Figures and Tables

**Table 1:** Physicomechanical properties of rock masses during the extraction of seam D in the Karaganda coal basin mines.

Parameter Type	Sandstone	Siltstone	Argillite	Coal
Compressive strength, MPa	30/60	33/45	15/30	4/10
Angle of internal friction, degrees	30/50	30/45	35/45	5/30
Tensile strength, MPa	5/20	8/20	2/5	1/2

## Data Availability

The raw data supporting the conclusions of this article will be made available by the authors on request.
